# Compressive stress-driven mechanosignaling in chondrocytes: From molecular mechanism to scaffold engineering and translational opportunities

**DOI:** 10.1016/j.mtbio.2026.103453

**Published:** 2026-07-14

**Authors:** Yongbing Mou, Tingting Tian, Peng Wang, Xia Wang, Wei Li, Wenfei Tang, Yehong Wang, Dong Zhu, Yong Huang, Xiao Huang

**Affiliations:** a School of Physical Education and Training, Xi'an Physical Education University, Xi'an, 710068, China; b Key Laboratory of Bone Biomaterials & Dong Medicine-Regulated Organoid Regeneration of Hunan Provincial Universities, Biomedical Research Institute, Hunan University of Medicine, Huaihua, 418000, China; c College of Rehabilitation Medicine and Health, Hunan University of Medicine, Huaihua, 418000, China; d College of Lab Medicine, Life Science Research Centre, Hebei North University, Zhangjiakou, 075000, China

**Keywords:** Compressive stress, Chondrocytes, Mechanotransduction, Cartilage repair, Osteoarthritis

## Abstract

Articular cartilage, a unique avascular and low-cell-density connective tissue, relies predominantly on chondrocyte responses to mechanical cues for the maintenance of tissue homeostasis and functional repair. Among the diverse mechanical stimuli encountered in the joint microenvironment, compressive stress stands as the most prominent and physiologically relevant physical signal regulating chondrocyte behavior. This review systematically dissects the multi-layered mechanisms underlying compressive stress-mediated chondrocyte regulation and its translational implications in cartilage tissue engineering and osteoarthritis (OA) intervention. At the molecular level, compressive stress initiates a cascade of mechanosensing, intracellular transduction, and functional output through the synergistic crosstalk of integrin-mediated adhesion complexes, calcium signaling networks, MAPK pathways, and downstream transcriptional regulators (e.g., SOX9, Runx2, Sp1), which collectively orchestrate the balance between anabolic and catabolic metabolism. At the cellular level, articular cartilage's inherent regional heterogeneity, coupled with distinct responses of healthy/pathological chondrocytes and stem cells to compressive parameters (frequency, strain magnitude, loading mode, duration), underscores the need for cell-type-specific mechanical intervention strategies. At the translational level, moderate dynamic compression promotes cartilage repair by preserving extracellular matrix integrity, suppressing inflammatory cascades, and modulating epigenetic landscapes, while aberrant loading exacerbates OA progression via chondrocyte apoptosis, matrix degradation, and pain sensitization. The optimization of scaffold materials (natural polymers, synthetic composites, intelligent responsive matrices) and culture systems (3D bioprinting, microfluidic bioreactors, shear-compression synergistic loading) has emerged as a critical enabler to enhance mechanical regulation efficacy. Despite significant advances, current research is constrained by insufficiently physiological in vitro/in vivo models, lack of standardized loading parameters, unclear pathway crosstalk mechanisms, and limited clinical translation of mechanical-based therapies. Future endeavors should prioritize the elucidation of multi-pathway synergistic networks using multi-omics approaches, establishment of personalized mechanical parameter databases integrating patient-specific factors (age, gender, disease severity), construction of bionic models recapitulating the joint's dynamic microenvironment, and development of combined mechanical-biological therapeutic strategies. These efforts will provide more precise molecular targets and clinically feasible schemes for cartilage repair and OA management.

## Graphical abstract

**Figure:**
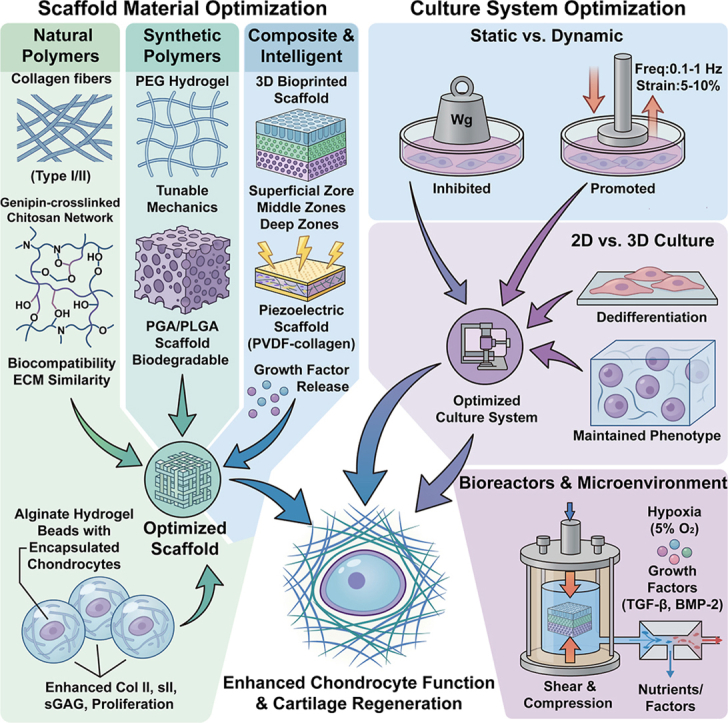

## 1 Introduction

Articular cartilage is a specialized tissue that underpins joint mobility by virtue of its unique biomechanical properties, conferred by an extracellular matrix (ECM) rich in type II collagen (Col II), aggrecan (ACAN), and sulfated glycosaminoglycans (sGAGs) [1]. As the sole cellular component of articular cartilage, chondrocytes are tasked with synthesizing and maintaining ECM homeostasis, a process that is tightly regulated by both biochemical and mechanical cues [2]. Unlike most tissues, articular cartilage lacks blood vessels, lymphatics, and nerves, rendering its self-repair capacity extremely limited [3]. Trauma, aging, obesity, and other risk factors disrupt chondrocyte phenotype and trigger ECM degradation, which constitutes the core pathological basis of degenerative joint diseases, most notably osteoarthritis (OA) [4]. OA affects over 300 million people worldwide, leading to chronic pain, joint stiffness, and functional disability, imposing a substantial burden on global healthcare systems [2].

However, the response of chondrocytes to mechanical loading is far from uniform across the joint surface, owing to the pronounced regional heterogeneity of articular cartilage. Regional heterogeneity of articular cartilage refers to the distinct structural, biochemical, and mechanical variations across different anatomical regions and depth zones of the tissue [5]. Superficially, chondrocytes are flattened and aligned parallel to the surface, providing shear resistance. In the middle zone, cells are rounded with high proteoglycan content for load distribution. Deep zone chondrocytes form columnar structures with vertically oriented collagen fibers to resist compression [[6], [7], [8]]. Such zonal and topographic differences adapt the cartilage to local mechanical environments, maintain joint function, and are closely associated with the onset and progression of osteoarthritis when disrupted.

Within this spatially heterogeneous cartilage tissue, several mechanical inputs coexist, including direct compressive stress, hydrostatic pressure generated by interstitial fluid pressurization, shear stress produced by relative tissue motion, and matrix deformation transmitted through the pericellular matrix and cytoskeleton. These stimuli are related but not interchangeable: hydrostatic pressure may occur with minimal cell shape change, whereas direct compression and matrix deformation more readily alter cell morphology, integrin engagement, membrane tension, and organelle mechanics [[9], [10], [11]]. In this review, “compressive stress” refers primarily to loading regimens that impose or transmit compressive deformation to cartilage, the pericellular matrix, and/or chondrocytes, while acknowledging that most experimental systems combine compressive, hydrostatic, and fluid-flow components to varying degrees.

In the complex microenvironment of chondrocytes, mechanical stimuli (including compression, shear, and tension) play pivotal roles in regulating cellular function. Among these, compressive stress is the dominant physical signal experienced by chondrocytes during joint movement, with physiological compressive loads ranging from 0.1 to 10 MPa in human joints [9]. Compressive stress exhibits a striking dual role in cartilage physiology and pathology [12]: Within the physiological range, moderate dynamic compression converts mechanical signals into intracellular biochemical cascades via mechanotransduction, promoting the synthesis of Col II, ACAN, and sGAGs while inhibiting the production of matrix-degrading enzymes (matrix metalloproteinases, MMPs; a disintegrin and metalloproteinase with thrombospondin motifs, ADAMTS) and pro-inflammatory cytokines (IL-1β, TNF-α) [3,13]. In contrast, abnormal mechanical stimuli, such as high-intensity static compression, impact loading, or joint immobilization, disrupt ECM structure, induce chondrocyte apoptosis and senescence, and accelerate cartilage degeneration, thereby driving OA initiation and progression [12,14].

The dual role of compressive stress highlights the critical importance of deciphering the molecular mechanisms by which chondrocytes sense and respond to mechanical signals, as well as optimizing mechanical intervention strategies for cartilage repair and OA treatment. Over the past decade, significant progress has been made in understanding the core components of compressive stress-medi ated mechanotransduction, including mechanosensors (integrins, ion channels, primary cilia), signaling pathways (calcium, MAPK, NAD-Sirtuin1-Runx2), and transcriptional regulators [15,16]. Additionally, advances in tissue engineering have led to the development of novel scaffold materials and culture systems that enhance the efficacy of mechanical regulation, bringing mechanical-based therapies closer to clinical application [17,18].

However, several key challenges remain unresolved: (1) In vitro models often fail to recapitulate the complex in vivo joint microenvironment, including multi-axial loading, dynamic inflammatory gradients, and cartilage-bone interface interactions; (2) Loading parameters (strain, frequency, duration) vary widely across studies, lacking standardization and hindering cross-study comparison; (3) The crosstalk between distinct mechanotransduction pathways and their integration with biochemical signals (e.g., cytokines, growth factors) are incompletely understood; (4) Clinical translation of mechanical-based therapies is limited by the lack of non-invasive delivery systems and personalized treatment protocols [12,19,20].

This review aims to provide a comprehensive and in-depth analysis of the current state of research on compressive stress-driven chondrocyte mechanotransduction. We first dissect the molecular mechanisms of mechanosensing, signal transduction, and functional regulation, emphasizing pathway crosstalk and epigenetic modulation. Next, we discuss cell-specific responses to compressive stress, including regional heterogeneity of articular cartilage and divergent behaviors of healthy/pathological chondrocytes and stem cells. We then explore the dual roles of compressive stress in cartilage repair and OA pathogenesis, followed by an overview of scaffold material optimization and culture system advancements. Finally, we highlight the current research bottlenecks and propose future directions to accelerate the translation of mechanical-based strategies into clinical practice. This review integrates the latest findings from molecular biology, tissue engineering, and clinical research, providing a holistic framework for understanding the role of compressive stress in chondrocyte biology and guiding the development of novel therapeutic interventions for cartilage-related diseases.

## 2 Molecular mechanisms of compressive stress-mediated mechanotransduction

**Fig. 1:**
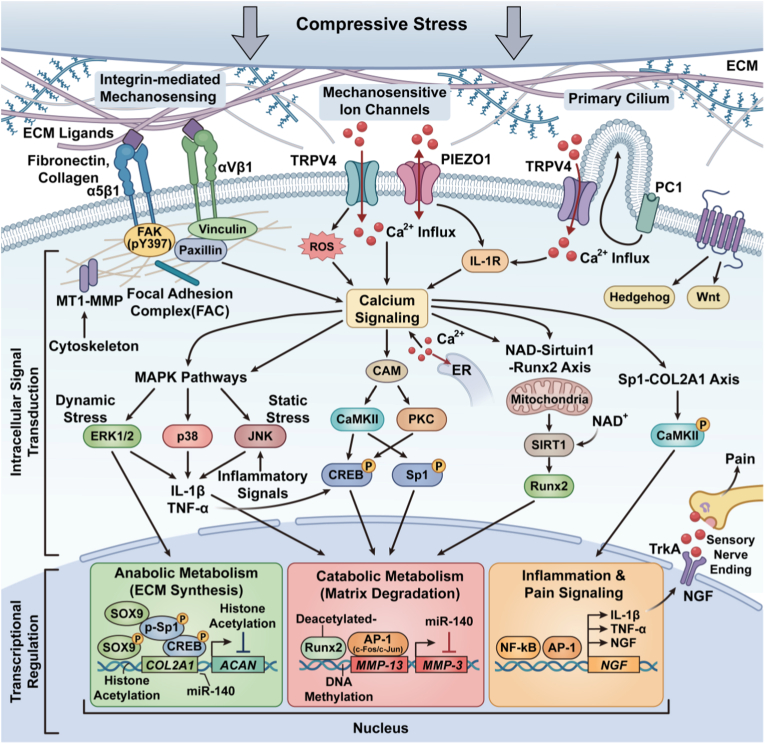
Schematic illustration of mechanotransduction mechanisms underlying chondrocyte responses to compressive stress.Compressive stress is transduced into chondrocytes via three primary mechanosensory modules at the cell-matrix interface: integrin-mediated focal adhesion signaling, mechanosensitive ion channels, and primary cilium-associated signaling (upper tier). These upstream modules converge on key intracellular signaling hubs, particularly calcium (Ca2^+^) signaling and MAPK pathways, alongside emergent force-sensing modules including mitochondrial mechanotransduction (NAD^+^-Sirtuin1-Runx2 axis), endoplasmic reticulum (ER) stress, and Hippo-YAP/TAZ-mediated nuclear mechanotransduction (middle tier). The transcriptional outputs of these signaling cascades are categorized into four major functional endpoints: anabolic metabolism (ECM synthesis), catabolic metabolism (matrix degradation), inflammatory and pain signaling, and cell fate regulation (lower tier). This framework prioritizes well-supported mechanistic axes supported by direct experimental evidence, while secondary or associative signaling modules (e.g., Hedgehog/Wnt pathways, epigenetic modifications) are included to highlight emerging concepts requiring further validation under defined compressive loading regimens.

Chondrocytes perceive extracellular compressive signals through specialized mechanosensors, transduce these signals into intracellular biochemical cascades, and ultimately regulate cellular functions such as ECM synthesis, metabolism, and survival. This mechanotransduction process involves a complex network of signaling pathways that act synergistically to orchestrate chondrocyte responses (Fig. 1). While the network is comprehensive, pathways differ markedly in the strength of supporting evidence: some are supported by direct, causal experimental data, whereas others remain associative or preliminary. Below, we dissect the key components of this network, from initial mechanosensing to downstream transcriptional regulation.

### 2.1 Mechanosensing: Initial perception of compressive stress

The first step in compressive stress-mediated mechanotransduction is the perception of mechanical signals by chondrocyte surface receptors and ECM-integrin complexes. These mechanosensors convert physical deformation of the ECM or cell membrane into biochemical signals that initiate downstream transduction.

#### 2.1.1 Integrin-mediated mechanosensing

Integrins, a family of heterodimeric transmembrane receptors (composed of α and β subunits), serve as the primary mechanosensors linking chondrocytes to the ECM [15]. This mechanism is supported by strong and direct evidence. Among the various integrin subtypes expressed by chondrocytes, α5β1 and αvβ1 are the most extensively studied in the context of compressive stress sensing. These integrins bind to ECM components such as fibronectin, collagen, and laminin, forming focal adhesion complexes (FACs) that include focal adhesion kinase (FAK), paxillin, and vinculin. When compressive stress is applied, ECM deformation induces conformational changes in integrins, leading to their clustering and activation of FAK via autophosphorylation at Tyr397 [21,22]. Activated FAK then phosphorylates downstream substrates such as paxillin, initiating signaling cascades that regulate cell spreading, cytoskeletal remodeling, and ECM metabolism. For example, Spiteri et al. [15] demonstrated that integrin-mediated cell spreading is critical for matrix accumulation during mechanical stimulation, and inhibition of integrin function abrogates compressive stress-induced proteoglycan synthesis. Additionally, integrins cooperate with membrane-type matrix metalloproteinase 1 (MT1-MMP) to regulate proteoglycan accumulation, forming a functional complex that modulates both ECM remodeling and mechanosensing [23,24].

#### 2.1.2 Mechanosensitive ion channels

Mechanosensitive ion channels, including transient receptor potential vanilloid 4 (TRPV4) and PIEZO1, play a crucial role in calcium influx triggered by compressive stress [16,25].This module is directly validated by gain-and loss-of-function studies. TRPV4, a non-selective cation channel, is highly expressed in chondrocytes and is activated by membrane stretch induced by compressive loading [26,27]. Activation of TRPV4 leads to rapid calcium influx, which serves as a second messenger to regulate downstream signaling pathways [28,29]. For instance, Takeda et al. [26] showed that cyclic compression induces reactive oxygen species (ROS) production in 3D-cultured ATDC5 cells, which upregulates IL-1 receptor (IL-1R) expression and enhances cell sensitivity to IL-1β. This process is regulated by TRPV4-mediated calcium signaling, as TRPV4 agonists inhibit catabolic gene expression, while antagonists enhance it [26]. PIEZO1, another mechanosensitive cation channel, has recently been identified as a key mediator of chondrocyte mechanotransduction. Nims et al. [16] demonstrated that activation of PIEZO1 by compressive stress drives unique transcriptional signatures in chondrocytes, including upregulation of genes involved in ECM synthesis and downregulation of pro-inflammatory factors. Notably, the activation of calcium signals by dynamic compression is independent of the number of loading cycles, suggesting that ion channels mediate rapid and direct responses to mechanical stimuli [28].

#### 2.1.3 Primary cilia

Primary cilia, microtubule-based organelles protruding from the chondrocyte surface, have emerged as important mechanosensors in recent years [9]. However, evidence for primary cilia remains largely associative, and direct causal mechanotransduction events are not fully defined. These structures are embedded in the ECM and are thought to detect mechanical deformation via their ciliary membrane receptors, including TRPV4 and polycystin-1 (PC1). When compressive stress is applied, bending of the primary cilium induces calcium influx and activates downstream signaling pathways such as Hedgehog and Wnt [30]. Although the exact role of primary cilia in compressive stress transduction remains incompletely defined, studies have shown that disruption of primary cilia function impairs chondrocyte responses to mechanical loading, leading to reduced ECM synthesis and increased catabolic activity.

#### 2.1.4 Cytoplasmic and intracellular force-sensing modules

Beyond classical surface mechanosensors, several intracellular structures are increasingly recognized as compression-responsive modules. These are emerging mechanisms with largely associative evidence; direct causal links are still being established. Mitochondria can undergo load-sensitive metabolic and redox changes that reshape ATP availability, ROS production, and catabolic signaling, particularly in OA settings [[31], [32], [33]]. Endoplasmic reticulum (ER) stress is also mechanically inducible and may link sustained compression to disturbed proteostasis, calcium dysregulation, and cartilage thinning [34]. In parallel, the nucleus and its LINC-complex-mediated cytoskeletal connections can function as a deformable signaling compartment, coupling changes in actomyosin tension to chromatin accessibility and transcriptional responses. These observations broaden mechanosensing from membrane-proximal receptors to an integrated cell-wide force-transmission system.

### 2.2 Intracellular signal transduction: Cascades mediating mechanical responses

Following initial mechanosensing, compressive stress signals are transduced intracellularly via a series of interconnected signaling pathways, including calcium signaling, MAPK pathways, and the NAD-Sirtuin1-Runx2 axis. These pathways work in concert to regulate chondrocyte metabolism and function.

#### 2.2.1 Calcium signaling: A central transduction hub

Calcium signaling is a ubiquitous and essential mediator of compressive stress-induced chondrocyte responses. This is a well-established, direct mechanotransduction hub. Compressive strain (10%–40%) activates calcium signaling in a gradient-dependent manner, with higher strains leading to more robust calcium transients [3]. Dynamic loading initiates calcium influx primarily through mechanosensitive ion channels (TRPV4, PIEZO1) and possibly via release from intracellular stores (endoplasmic reticulum) [28]. The spatial distribution of ECM strain also influences calcium signaling, with more pronounced effects observed in patellofemoral joint cartilage compared to other regions [35]. Calcium influx acts as a second messenger to regulate downstream targets, including calmodulin (CaM), calcium/calmodulin-dependent protein kinases (CaMKs), and protein kinase C (PKC) [36]. For example, calcium-CaM complexes activate CaMKII, which phosphorylates transcription factors such as CREB, leading to the upregulation of anabolic genes including COL2A1 and ACAN. Additionally, calcium signaling regulates the early stages of cartilage tissue formation induced by compressive stress, as inhibition of calcium influx abrogates compression-mediated matrix synthesis [37].

#### 2.2.2 MAPK pathways: Integrating mechanical and inflammatory signals

The mitogen-activated protein kinase (MAPK) pathway (comprising p38, c-Jun N-terminal kinase (JNK), and extracellular signal-regulated kinase (ERK1/2)) serves as a key hub for integrating mechanical signals with inflammatory cues [38].MAPK pathways are directly validated; specific inhibition abolishes compression-driven metabolic control. Cyclic hydrostatic pressure, a form of compressive stress, inhibits the phosphorylation of p38, JNK, and ERK1/2, reversing IL-1β-induced suppression of chondroprogenitor cell proliferation and downregulating matrix-degrading enzymes such as MMP-13 and ADAMTS5 [39]. Fitzgerald et al. [10] demonstrated that sustained activation of ERK1/2 and delayed activation of p38 are required for shear- and compression-induced transcription of aggrecan, Col II, and MMP-13 in cartilage explants. Moreover, MEK/ERK pathway inhibitors and p38 kinase inhibitors block these effects, confirming the critical role of MAPK kinases in signal conversion. The specificity of MAPK pathway activation depends on the nature of the compressive stress: dynamic compression predominantly activates ERK1/2 to promote anabolic metabolism, while static compression enhances p38 and JNK phosphorylation to induce catabolic responses.

#### 2.2.3 Hippo-YAP/TAZ and nuclear mechanotransduction

YAP/TAZ has emerged as an important but context-sensitive mediator of chondrocyte mechanobiology. Evidence for YAP/TAZ in compressive mechanotransduction remains associative rather than universally causal. In highly spread or stiff microenvironments, YAP/TAZ activation is often associated with suppression of chondrogenic gene expression, cytoskeletal tension, and phenotypic drift away from a stable cartilage program [40]. However, YAP signaling may also restrain selected inflammatory or breakdown pathways under specific OA-related conditions, indicating that its role cannot be reduced to a simple anabolic-versus-catabolic binary [41,42]. For compressive stress studies, the critical issue is whether loading promotes physiologic, rounded, PCM-buffered mechanotransduction or instead drives nuclear tension and mechanoactivation associated with dedifferentiation. This makes YAP/TAZ especially relevant for interpreting differences between 2D expansion systems, 3D embedded culture, and scaffold designs with distinct confinement and stiffness profiles.

#### 2.2.4 NAD-Sirtuin1-Runx2 axis: Regulating mitochondrial function and catabolism

Repetitive compressive loading activates the NAD-dependent deacetylase Sirtuin1 (SIRT1) and Runt-related transcription factor 2 (Runx2), forming a signaling axis that regulates mitochondrial function and matrix degradation [43]. This axis is preliminary and largely disease-specific; direct evidence in healthy cartilage is limited. Takemoto et al. [31] showed that repetitive compressive loading downregulates mitochondrial ATP production by consuming mitochondrial NAD, which activates SIRT1. Activated SIRT1 then deacetylates Runx2, enhancing its transcriptional activity and promoting the expression of MMP-13, a key matrix-degrading enzyme in OA [44,45]. This pathway plays a critical role in OA progression, as inhibition of SIRT1 or Runx2 abrogates compression-induced MMP-13 upregulation and mitochondrial dysfunction. Notably, this axis is selectively activated in OA chondrocytes, suggesting that it may represent a disease-specific therapeutic target.

#### 2.2.5 Sp1-COL2A1 axis: Promoting anabolic metabolism

Dynamic compression enhances Col II synthesis by activating the proximal Sp1 binding site of the COL2A1 gene promoter [46]. This pathway is partially supported, but in vivo physiological confirmation remains incomplete. Mechanical compressive loading increases Sp1 phosphorylation and nuclear translocation in chondrocytes, leading to enhanced binding of Sp1 to the COL2A1 promoter and increased transcriptional activity. This pathway is synergistically regulated by calcium signaling, as calcium influx enhances Sp1 phosphorylation via CaMKII [47]. Additionally, SOX9, a master regulator of chondrogenesis, cooperates with Sp1 to activate COL2A1 transcription, forming a complex network that promotes anabolic metabolism [48].

### 2.3 Transcriptional regulation and functional output

The convergence of intracellular signaling pathways ultimately regulates the expression of target genes involved in anabolic metabolism, catabolism, inflammation, and cell survival. Key transcriptional regulators include SOX9, Runx2, Sp1, and activator protein-1 (AP-1), which collectively orchestrate the balance between cartilage homeostasis and degeneration.

#### 2.3.1 Anabolic regulation: SOX9 and Sp1

SOX9 is a transcription factor essential for chondrocyte differentiation and ECM synthesis. SOX9 is a directly validated anabolic regulator under compressive loading. Dynamic compression upregulates SOX9 expression and nuclear localization in OA chondrocytes, leading to enhanced transcription of COL2A1 and ACAN [49,50]. Dynamic cyclic compression modulates the chondrogenic phenotype of late-stage OA chondrocytes by upregulating SOX9, which inhibits collagen degradation and enhances cartilage-specific gene expression [48]. Sp1, as discussed earlier, directly activates COL2A1 transcription and cooperates with SOX9 to amplify anabolic responses [47]. Together, SOX9 and Sp1 form a central transcriptional module that mediates compression-induced matrix synthesis.

#### 2.3.2 Catabolic regulation: Runx2 and AP-1

Runx2, traditionally known for its role in osteogenesis, also promotes catabolic metabolism in chondrocytes. Causal links are partially validated and stronger under pathological loading. Compression-induced activation of Runx2 upregulates MMP-13 and downregulates Col II expression, contributing to matrix degradation [31]. AP-1, a dimeric transcription factor composed of c-Fos and c-Jun subunits, is another key mediator of catabolic responses. Cyclic compressive stress induces sequential expression of catabolic genes (MMP-3, MMP-13) and anabolic genes (Col II, aggrecan) in chondrocytes, a process dependent on MAPK-mediated activation of AP-1 [51]. The temporal regulation of AP-1 activity may explain the biphasic responses of chondrocytes to mechanical loading [52].

#### 2.3.3 Inflammatory regulation: NGF and cytokine networks

Compressive stress also regulates inflammatory responses and pain signaling in chondrocytes. Evidence linking compression to NGF-mediated pain is associative; direct mechanistic cascades require further validation. Compressive stress synergizes with IL-1β and visfatin to induce nerve growth factor (NGF) expression and release in chondrocytes [53,54]. NGF binds to tropomyosin receptor kinase A (TrkA) on sensory nerve endings, initiating pain signaling and contributing to OA-related pain [55]. Additionally, dynamic compression inhibits the production of pro-inflammatory cytokines (IL-1β, TNF-α) and chemokines (IL-8) in chondrocytes, while static compression enhances their release [56,57]. This regulation is mediated by MAPK and NF-κB pathways, as compression modulates the phosphorylation and nuclear translocation of NF-κB p65 [57].

### 2.4 Pathway crosstalk and epigenetic modulation

The mechanotransduction network is further complicated by extensive crosstalk between signaling pathways and epigenetic regulation. Most observations of crosstalk and epigenetic modulation remain associative; direct hierarchical causal mechanisms are incompletely resolved. For example, integrin activation induces calcium influx, which in turn activates MAPK pathways, forming a positive feedback loop that amplifies mechanical signals [3,15]. TRPV4-mediated calcium signaling regulates the ROS-IL-1R pathway, linking mechanical stress to inflammatory responses [26]. Additionally, mechanical stress modulates epigenetic modifications such as DNA methylation, histone acetylation, and microRNA (miRNA) expression, which contribute to long-term regulation of chondrocyte phenotype [58]. For instance, compressive stress increases the acetylation of histone H3 at the COL2A1 promoter, enhancing its transcriptional activity. Conversely, OA chondrocytes exhibit hypermethylation of the COL2A1 promoter and hypomethylation of the MMP-13 promoter, which are exacerbated by abnormal mechanical loading [59]. miRNAs such as miR-140, a cartilage-specific miRNA, are also regulated by compressive stress: dynamic compression upregulates miR-140, which inhibits MMP-13 and ADAMTS5 expression, while static compression downregulates miR-140 [19]. These epigenetic mechanisms provide a molecular basis for the long-term effects of mechanical stress on chondrocyte function and cartilage homeostasis.

## 3 Cell-specific responses to Compressive Stress

**Fig. 2:**
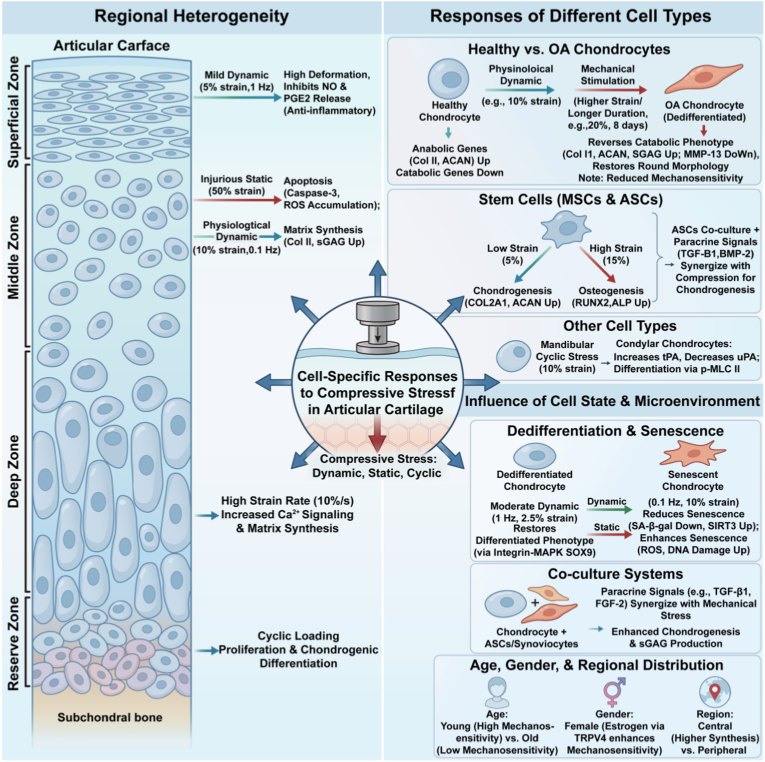
Schematic Depiction of Cell-Specific Responses to Compressive Stress in Articular Cartilage: Regional Heterogeneity and Microenvironmental Modulation This schematic illustrates a mechanotransduction framework centered on compressive stress (dynamic, static, cyclic) as the core upstream signal, integrating tissue-scale spatial heterogeneity, cell-type specific responsiveness, and contextual regulators to drive articular cartilage homeostasis. Central Signaling Input Module Compressive stress, defined by magnitude (strain %), mode (dynamic/static/cyclic), frequency (Hz), and duration, acts as the primary mechanical trigger. This upstream module modulates downstream signaling cascades across distinct spatial and cellular compartments. **Left Panel:** Regional Heterogeneity in Mechanical Responsiveness Articular cartilage exhibits zonal-dependent mechanoresponse due to differential cell morphology, matrix composition, and metabolic states: Superficial Zone (SZ): Mild dynamic loading (5% strain, 1 Hz) elicits anti-inflammatory responses (inhibited NO/PGE_2_ release), whereas high-deformation static loading (50% strain) induces apoptosis via Caspase-3/ROS accumulation. Middle Zone (MZ): Physiologically dynamic loading (10% strain, 0.1 Hz) promotes matrix synthesis (sGAG upregulation) and maintains chondrocyte anabolism. Deep Zone (DZ) & Reserve Zone: High-strain conditions (10% strain) enhance Ca^2+^ signaling and matrix synthesis; cyclic loading drives chondrocyte proliferation and chondrogenic differentiation. **Right Upper Panel:** Cell-Type Specific Responses Distinct cell populations exhibit divergent mechanophenotypes: Healthy vs. OA Chondrocytes: Physiological dynamic loading (10% strain) maintains healthy chondrocyte anabolism (Col II, ACAN up) and catabolic gene suppression. In contrast, pathological mechanical stimuli (higher strain, longer duration) induce dedifferentiation in OA chondrocytes (Col I, ACAN, SOX9 up; MMP-13 down), reverse their catabolic phenotype, restore round morphology, and reduce mechanosensitivity. Mesenchymal Stem Cells (MSCs) & Adipose-Derived Stem Cells (ASCs): Low-strain conditions promote chondrogenesis (Col2A1, ACAN, RUNX2 down); high-strain conditions shift differentiation toward osteogenesis (ALP up). Paracrine signals (e.g., TGF-β1, BMP-2) synergize with mechanical strain to enhance chondrogenic commitment. Other Cell Types: Condylar chondrocytes respond to cyclic stress (10% strain) via mandibular condyle growth modulation (IPA upregulation) and differentiation via p-MLC II signaling. **Right Lower Panel:** Modulation by Cell State & Microenvironment Cell state and contextual factors refine mechanical responses: Dedifferentiation & Senescence: Dedifferentiated chondrocytes exhibit reduced mechanosensitivity; moderate dynamic loading (1 Hz, 2.5% strain) restores their differentiated phenotype (via integrin-MAPK signaling). Senescent chondrocytes (10% strain) show reduced senescence (SA-β-gal down, SIRT3 up) but enhanced DNA damage (ROS up) under static conditions. Co-Culture Systems: Chondrocyte-stem cell/synoviocyte co-cultures enhance chondrogenesis and sGAG production via paracrine crosstalk (e.g., TGF-β1, FGF-2) synergizing with mechanical stress. Demographic & Regional Modifiers: Age (high mechanosensitivity in youth), gender (estrogen via TRPV4 in females), and regional distribution (higher synthesis in central vs. peripheral regions) further stratify mechanical responsiveness.

Chondrocyte responses to compressive stress are highly context-dependent, shaped by articular cartilage's inherent regional heterogeneity, cell type (healthy vs. pathological chondrocytes, stem cells), and functional state (dedifferentiated, senescent, co-cultured) (Fig. 2). Understanding these cell-specific responses is critical for designing targeted mechanical intervention strategies for cartilage repair and OA treatment.

### 3.1 Regional heterogeneity of articular cartilage

Articular cartilage is divided into three distinct zones, superficial (tangential), middle (transitional), and deep (radial), including the reserve zone. Each zone is characterized by unique chondrocyte morphology, ECM composition, and mechanical properties [60]. This regional heterogeneity translates into distinct responses to compressive stress.

This framework delineates the hierarchical axis: mechanical stress input—zonal/cellular mechanotransduction—microenvironmental regulation, providing a systemic model for cartilage mechanobiology and OA pathogenesis.

#### 3.1.1 Superficial zone chondrocytes

Superficial zone chondrocytes are flattened, elongated cells arranged parallel to the articular surface, with a high cell density and low ECM content [60,61]. These cells express superficial zone protein (SZP), a glycoprotein that reduces friction and enhances lubrication in the joint [62]. Physiological dynamic compression inhibits the release of nitric oxide (NO) and prostaglandin E2 (PGE2) from superficial zone chondrocytes, counteracting the pro-inflammatory effects of IL-1β [63]. Komeili et al. [60] used multiphoton excitation microscopy to demonstrate that superficial zone cells exhibit greater deformation amplitude (up to 30% strain) than middle zone cells under mild dynamic loading (5% strain, 1 Hz), with smaller volume loss compared to static compression. This enhanced deformability may be attributed to the high cytoskeletal flexibility of superficial zone chondrocytes, allowing them to adapt to repetitive mechanical loading. Additionally, superficial zone chondrocytes show greater sensitivity to shear stress, which may cooperate with compressive stress to regulate tissue homeostasis [63].

#### 3.1.2 Middle zone chondrocytes

Middle zone chondrocytes are rounded or polygonal cells arranged randomly, with a moderate cell density and abundant ECM [60]. This zone is the thickest region of articular cartilage and bears the brunt of mechanical loading during joint movement. Middle zone chondrocytes are more susceptible to apoptosis under damaging compressive stress (e.g., 50% strain, static loading) compared to other zones. Injurious mechanical compression induces chondrocyte apoptosis in the middle zone via caspase-3 activation and ROS accumulation, a process that contributes to cartilage degeneration [64]. However, physiological dynamic compression (10% strain, 0.1 Hz) promotes matrix synthesis in middle zone chondrocytes, increasing Col II and sGAG production [65]. The biphasic response of middle zone chondrocytes to compressive stress highlights the importance of loading parameters in determining cellular outcomes.

#### 3.1.3 Deep zone and reserve zone chondrocytes

Deep zone chondrocytes are large, rounded cells arranged perpendicular to the articular surface, with a low cell density and high proteoglycan content [60]. The reserve zone, located adjacent to the subchondral bone, contains chondroprogenitor cells that contribute to cartilage maintenance and repair. The mechanical response of deep zone chondrocytes is regulated by tissue depth and strain rate: higher strain rates (10%/s) induce greater calcium signaling and matrix synthesis compared to lower strain rates (1%/s) [66]. Reserve zone chondrocytes exhibit depth-dependent mechanical responses, with cells closer to the subchondral bone showing reduced deformation and calcium influx under compressive loading. This regional specificity may be due to differences in ECM stiffness and integrin expression between deep zone and reserve zone chondrocytes. Additionally, cyclic compressive loading promotes the proliferation and chondrogenic differentiation of reserve zone chondroprogenitor cells, suggesting a role in cartilage regeneration [66].

### 3.2 Responses of different cell types

Different chondrocyte subtypes exhibit distinct responses to compressive stress, and their responses to various parameters also differ (Table 1).

**Table 1:** Representative compression regimens across experimental contexts and their major biological outputs.

Experimental context	Predominant loading mode	Representative parameter window	Main biological readouts	Interpretive note	Key cross-study comparison insights	Ref
Native cartilage explants/primary chondrocytes	Moderate dynamic compression	Low-to-moderate strain or pressure; low frequency; recovery permitted	↑ Col II, ACAN, sGAG; ↓ IL-1-induced catabolic signaling	Most reproducible anabolic window, but species, depth, and waveform still matter	**Horizontal comparison:** Anabolic responses to 3–5% dynamic compression are consistently stronger in bovine/porcine chondrocytes than in rat/murine cells. Superficial zone chondrocytes are more sensitive to 0.1–0.5 Hz loading, while mid-zone cells respond better to 0.5–1 Hz, indicating depth-dependent mechanosensitivity across studies.	[67,68]
OA chondrocytes	Intermittent cyclic compression	Often requires longer conditioning and/or recalibrated strain amplitude	Partial redifferentiation; ↑ COL2A1 and ACAN; ↓ MMP-13	Disease state shifts the beneficial window relative to healthy cells	**Horizontal comparison:** The optimal strain window for OA chondrocytes (1–3%) is markedly narrower than for healthy chondrocytes (3–10%). Intermittent loading consistently outperforms continuous cyclic loading in suppressing catabolism, with consistent trends across independent models.	[31,68,69]
MSC/ASC-based constructs	Dynamic compression in 3D scaffolds	Low strain favors chondrogenesis; higher strain may bias osteogenesis	Lineage specification, matrix deposition, growth-factor sensitivity	Cell source and scaffold confinement strongly modify the response	**Horizontal comparison:** BMSCs exhibit higher chondrogenic efficiency than ASCs under identical low-strain compression. Hydrogel-encapsulated MSCs favor chondrogenesis at <5% strain, whereas porous scaffolds promote osteogenic bias at >8% strain, revealing scaffold-dependent lineage switching.	[68,[70], [71], [72]]
Explant or chip-based disease models	Programmable dynamic or hyperphysiological compression	Used to mimic OA-like overload or to quantify inflammatory outputs in real time	NO release, inflammatory genes, matrix injury markers	High physiologic relevance, but cross-platform standardization remains limited	**Horizontal comparison:** Hyperphysiological compression (10–15%) induces 3–5-fold higher NO release in explants than physiological loading. Chip-based models show even stronger inflammatory responses due to integrated cytokine microenvironments, yet poor standardization of strain, frequency, and sensing limits direct quantitative comparison.	[31,68,73,74]

#### 3.2.1 Healthy and OA chondrocytes

Healthy and OA chondrocytes exhibit distinct responses to compressive stress, reflecting the phenotypic changes associated with OA progression. Healthy chondrocytes maintain a stable chondrogenic phenotype and respond to physiological dynamic compression by upregulating anabolic genes and downregulating catabolic genes. In contrast, OA chondrocytes exhibit a dedifferentiated phenotype, with reduced Col II and aggrecan expression and increased MMP-13 and ADAMTS5 expression [75,76]. However, mechanical stimulation can partially reverse the catabolic phenotype of OA chondrocytes [77]. Intermediate-term intermittent cyclic sinusoidal compression (1 Hz, 2.5% maximum amplitude, 4 days) significantly upregulates COL2A1 and ACAN gene expression in human dedifferentiated OA chondrocytes, downregulates MMP-13, increases sGAG content by 35%, and restores cell morphology from spindle-shaped to round [78]. Chong et al. [79] further confirmed that dynamic compression (10%–20% strain, 4–8 days) promotes OA chondrocytes to express cartilage-specific markers such as biglycan (BGN), CD90, and heparan sulfate proteoglycan 2 (HSPG-2), with the 20% strain, 8-day loading group showing a 2.3-fold increase in type VI collagen fluorescence intensity compared to unloaded controls. These findings suggest that mechanical stimulation may be a viable strategy to restore the functional phenotype of OA chondrocytes, expanding the indications of matrix-assisted autologous chondrocyte implantation (MACI) technology.

Notably, the optimal compression parameters for OA chondrocytes differ from those for healthy chondrocytes. For example, healthy chondrocytes show maximal anabolic responses to 10% strain, 1 Hz dynamic compression, while OA chondrocytes require higher strain (20%) and longer duration (8 days) to achieve similar effects [65,79]. This difference may be due to reduced mechanosensitivity of OA chondrocytes, attributed to downregulation of integrins and mechanosensitive ion channels [16]. Single-cell RNA sequencing studies have further revealed that OA chondrocytes exhibit heterogeneous responses to mechanical stimulation, with a subset of cells showing robust anabolic responses and another subset remaining in a catabolic state [21]. This heterogeneity highlights the need for personalized mechanical intervention strategies that account for disease severity and cellular subpopulations.

#### 3.2.2 Stem cells: MSCs and ASCs

Mesenchymal stem cells (MSCs) and adipose-derived stem cells (ASCs) are promising cell sources for cartilage tissue engineering due to their ability to differentiate into chondrocytes [80,81]. Compressive stress plays a critical role in regulating the chondrogenic differentiation of these stem cells. Horner et al. [82] showed that dynamic compressive strain induces magnitude-dependent and inversely related osteogenic/chondrogenic differentiation of human MSCs: low strain (5%) promotes chondrogenesis (upregulation of COL2A1, ACAN), while high strain (15%) enhances osteogenesis (upregulation of RUNX2, ALP). Cyclic compression (1 Hz, 10% strain) induces the formation of a mature meniscal cell phenotype in MSCs seeded on atelocollagen-based scaffolds, as evidenced by upregulation of meniscal-specific markers (type I collagen, aggrecan) and improved biomechanical properties [83]. Additionally, the combination of dynamic culture and cyclic compression promotes MSC proliferation by 40% and chondrogenesis by 60% compared to static culture [84].

ASCs, like MSCs, exhibit chondrogenic responses to compressive stress. A compression protocol of 20% strain, 3 h/day is optimal for porcine chondrocytes and ASCs co-cultured in cryogel scaffolds: this parameter upregulates Col II, TGF-β1, and IGF-1 expression in chondrocytes and promotes chondrogenic differentiation of ASCs [70]. Notably, ASCs can replace 50% of chondrocytes in the co-culture system without impairing ECM production, suggesting a cost-effective strategy for cartilage tissue engineering. The chondrogenic differentiation of ASCs is also regulated by paracrine signals: ASCs secrete TGF-β1 and BMP-2, which synergize with compressive stress to enhance chondrogenesis [70].

#### 3.2.3 Other cell types

Rat mandibular condylar chondrocytes, which are involved in temporomandibular joint function, exhibit distinct responses to cyclic compressive stress. Chen et al. [85] showed that cyclic mechanical stress regulates plasminogen activator activity and expression in these cells, with 10% strain, 0.5 Hz compression increasing tissue-type plasminogen activator (tPA) expression by 2.1-fold and decreasing urokinase-type plasminogen activator (uPA) expression by 40%. Another study further demonstrated that cyclic compressive stress induces the differentiation of rat primary mandibular condylar chondrocytes via phosphorylated myosin light chain II (p-MLC II), as inhibition of MLC kinase abrogates compression-mediated upregulation of Col II and aggrecan [86].

### 3.3 Influence of cell state and microenvironment

#### 3.3.1 Dedifferentiation and senescence

Chondrocytes undergo dedifferentiation during in vitro culture, characterized by loss of the chondrogenic phenotype (downregulation of Col II, upregulation of Col I) and reduced matrix synthesis. Moderate dynamic compression (1 Hz, 2.5% strain) restores the differentiated phenotype of dedifferentiated chondrocytes. This restoration is mediated by integrin-MAPK-SOX9 signaling, as inhibition of integrins or MAPK pathways abrogates the effects of mechanical stimulation [78].

Chondrocyte senescence, a hallmark of aging and OA, is also regulated by compressive stress. Senescent chondrocytes exhibit increased expression of p16INK4a and p21, reduced proliferation, and enhanced catabolic activity. Dynamic compression (0.1 Hz, 10% strain) reduces senescence-associated β-galactosidase (SA-β-gal) activity in chondrocytes by 30% and upregulates the expression of sirtuin 3 (SIRT3), a mitochondrial deacetylase that inhibits senescence. In contrast, static compression enhances chondrocyte senescence by increasing ROS production and DNA damage [2].

#### 3.3.2 Co-culture systems

Co-culture of chondrocytes with stem cells (MSCs, ASCs) or other cell types (synoviocytes) modulates their responses to compressive stress via paracrine signaling. In chondrocyte-ASC co-cultures, paracrine factors such as TGF-β1, BMP-2, and IL-6 synergize with mechanical stress to promote chondrogenesis. The paracrine signals from ASCs enhance the anabolic responses of chondrocytes to compression, while chondrocytes secrete factors that promote the chondrogenic differentiation of ASCs [70]. Similarly, co-culture of chondrocytes with synoviocytes under dynamic compression increases sGAG production by 50% compared to chondrocyte monoculture, attributed to synoviocyte-derived growth factors (e.g., FGF-2) [87].

#### 3.3.3 Age, gender, and regional distribution

Cell age significantly influences chondrocyte responses to compressive stress. Dynamic compressive loading differentially regulates anabolic and catabolic activity in chondrocytes from young (3 months) and old (24 months) rats: young chondrocytes exhibit a 40% increase in sGAG synthesis under 10% strain, 1 Hz compression, while old chondrocytes show only a 15% increase. This age-related decline in mechanosensitivity is associated with downregulation of integrins and calcium channels [88].

Gender also plays a role in chondrocyte mechanical responses. Female chondrocytes exhibit higher MMP-13 expression and lower Col II expression under static compression compared to male chondrocytes, possibly due to differences in estrogen signaling. Estrogen enhances chondrocyte mechanosensitivity by upregulating TRPV4 expression, suggesting a protective role in females [87].

Chondrocytes from different regions of the joint also show distinct responses to dynamic compression. Central region tibial plateau chondrocytes exhibit a 30% higher sGAG synthesis rate under dynamic compression compared to peripheral region chondrocytes. This regional difference is attributed to variations in ECM composition and integrin expression [87].

## 4 Roles of compressive stress in cartilage repair and OA pathogenesis

**Fig. 3:**
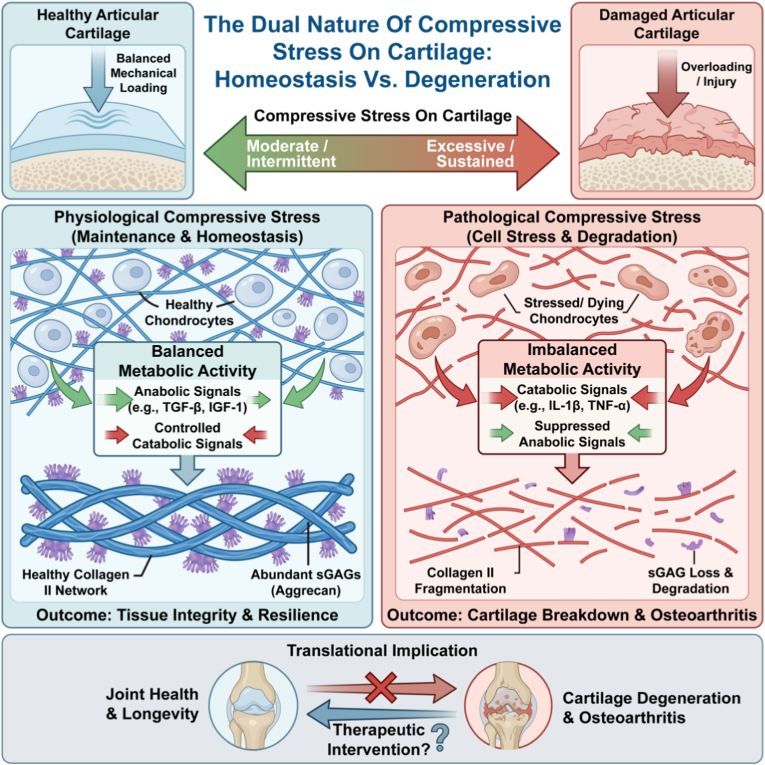
The dual nature of compressive stress on articular cartilage: homeostasis versus degeneration.Schematic illustration depicting the dichotomous effects of compressive stress on articular cartilage, structured around a spectrum of mechanical loading intensities, distinct cellular and molecular responses, and consequent tissue outcomes. At the top, a gradient of compressive stress spans from moderate/intermittent (left, associated with healthy cartilage) to excessive/sustained (right, linked to damaged cartilage). The left panel elaborates on physiological compressive stress, wherein healthy chondrocytes maintain balanced metabolic activity, characterized by tightly regulated anabolic signals (e.g., TGF-β, IGF-1) and controlled catabolic signals. This cellular homeostasis sustains the integrity of the collagen II network and abundant sGAGs (aggrecan), ultimately preserving tissue integrity and resilience. Conversely, the right panel outlines pathological compressive stress, where stressed or dying chondrocytes exhibit imbalanced metabolic activity: aberrantly upregulated catabolic signals (e.g., IL-1β, TNF-α) coincide with suppressed anabolic signals. This metabolic dysregulation drives collagen II fragmentation and sGAG loss/degradation, culminating in cartilage breakdown and osteoarthritis. At the bottom, the translational implication section highlights the clinical relevance of these mechanisms, delineating the transition from joint health and longevity to cartilage degeneration and osteoarthritis, and identifying potential targets for therapeutic intervention. The framework establishes a mechanistic link between mechanical loading, cellular metabolism, extracellular matrix remodeling, and joint tissue fate, providing a systematic blueprint for understanding cartilage homeostasis and degeneration.

Compressive stress exerts dual and context-dependent effects on cartilage homeostasis: physiological dynamic compression promotes tissue repair and maintenance, while abnormal loading (excessive compression or unloading) drives OA progression (Fig. 3). These effects are mediated by regulation of ECM metabolism, inflammatory responses, pain signaling, and epigenetic landscapes.

### 4.1 Physiological compressive stress: Promoting cartilage repair and homeostasis

Moderate dynamic compression within the physiological range (0.1–10 MPa, 0.01–1 Hz, 1%–10% strain) is essential for maintaining cartilage homeostasis and promoting repair [65]. This form of mechanical stimulation regulates multiple aspects of chondrocyte function to preserve ECM integrity.

#### 4.1.1 Enhancing anabolic metabolism

Dynamic compression stimulates the synthesis of key ECM components, including Col II, aggrecan, and sGAGs. Dynamic compression at 0.01–1 Hz and 1%–5% strain increases 3H-proline (Col II precursor) and 35S-sulfate (sGAG precursor) incorporation in cartilage explants by 20%–40% [65]. The anabolic effect of dynamic compression is mediated by SOX9 and Sp1, as discussed earlier, and is synergistically enhanced by growth factors such as TGF-β3. Delayed compressive loading (applied on day 7 of culture) has a beneficial effect on TGF-β3-cultured tissue-engineered cartilage constructs, increasing sGAG content by 50% and improving compressive modulus by 30% compared to immediate loading [89].

#### 4.1.2 Inhibiting catabolic activity

Dynamic compression inhibits the production of matrix-degrading enzymes (MMPs, ADAMTS) and pro-inflammatory cytokines (IL-1β, TNF-α) in chondrocytes. Mechanical loading of articular cartilage reduces IL-1-induced expression of MMP-1, MMP-3, and MMP-13 by 40%–60%. This inhibition is mediated by MAPK pathways: dynamic compression downregulates p38 and JNK phosphorylation, which are required for IL-1-induced catabolic gene expression [90,91]. Additionally, dynamic compression upregulates tissue inhibitor of metalloproteinases (TIMP-1, TIMP-3), which bind to MMPs and inhibit their activity [56].

#### 4.1.3 Modulating epigenetic landscapes

Physiological compressive stress modulates epigenetic modifications to promote long-term cartilage homeostasis. Dynamic compression increases histone H3 acetylation at the COL2A1 promoter by 2.5-fold, enhancing its transcriptional activity [59]. Compression also upregulates miR-140, a cartilage-specific miRNA that targets MMP-13 and ADAMTS5. miR-140 overexpression mimics the anabolic effects of dynamic compression, while miR-140 inhibition abrogates them [19]. These epigenetic changes contribute to the sustained maintenance of the chondrogenic phenotype.

### 4.2 Abnormal compressive stress: Driving OA pathogenesis

Abnormal mechanical stimuli (high-intensity static compression, impact loading, and joint immobilization) disrupt cartilage homeostasis and accelerate OA progression via multiple mechanisms. Notably, abnormal compressive stress does not act in isolation but integrates with oxidative stress amplification, inflammatory signaling, chondrocyte senescence, and mitochondrial dysfunction to form a self-reinforcing pathological cascade that extends far beyond matrix breakdown.

#### 4.2.1 Inducing chondrocyte apoptosis and necrosis

Excessive compressive stress (e.g., 20%–50% strain, static loading) directly damages the ECM structure and induces chondrocyte apoptosis [64]. Mechanical injury to cartilage induces chondrocyte apoptosis via caspase-3 activation and ROS accumulation, with 50% strain static compression increasing apoptosis rate by 3.2-fold compared to unloaded controls [92]. The apoptotic response is mediated by the mitochondrial pathway: excessive compression disrupts mitochondrial membrane potential, leading to release of cytochrome *c* and activation of caspases. In addition to apoptosis, high-intensity impact loading induces chondrocyte necrosis, characterized by cell membrane rupture and release of intracellular contents, which further exacerbates inflammation [14].

#### 4.2.2 Enhancing matrix degradation

Abnormal compressive stress upregulates the expression and activity of matrix-degrading enzymes, leading to ECM degradation. Repetitive compressive loading upregulates MMP-13 expression in OA chondrocytes via the NAD-Sirtuin1-Runx2 axis, as discussed earlier [31]. Static compression (3 MPa) increases MMP-1, MMP-3, and MMP-13 expression by 2.3–3.1-fold in chondrocytes, while decreasing Col II and aggrecan expression by 40%–50% [93]. This catabolic response is mediated by NF-κB pathway activation: static compression increases the phosphorylation and nuclear translocation of NF-κB p65, which binds to the promoters of MMP genes and enhances their transcription [57].

#### 4.2.3 Promoting inflammation and pain

Abnormal mechanical loading enhances the production of pro-inflammatory cytokines and pain mediators in chondrocytes. This process is further amplified by compression-triggered mitochondrial dysfunction and ROS accumulation, which lower the threshold for inflammatory activation. Static compression (4 MPa) increases IL-1β and TNF-α expression by 2.8-fold and 3.5-fold, respectively, in chondrocytes. These cytokines further amplify catabolic responses by upregulating MMPs and inhibiting ECM synthesis [57]. Compressive stress also induces NGF expression in chondrocytes, which contributes to OA pain. NGF binds to TrkA on sensory nerve endings, initiating the release of substance P and calcitonin gene-related peptide (CGRP), which mediate pain perception. Inhibition of NGF-TrkA signaling reduces OA-related pain in animal models, highlighting the therapeutic potential of targeting this pathway [55].

#### 4.2.4 Accelerating chondrocyte senescence and epigenetic disruption disrupting epigenetic regulation

Chronic aberrant compression also promotes chondrocyte senescence and mitochondrial impairment, which reinforce a degenerative phenotype independently of matrix metabolism. Static or excessive loading elevates ROS-mediated DNA damage, upregulates p16INK4a and p21, and increases SA-β-gal activity, pushing chondrocytes into a persistent senescent state. Senescent cells secrete pro-inflammatory and catabolic factors (SASP), creating a local microenvironment that perpetuates degeneration.

Abnormal mechanical loading alters epigenetic modifications in chondrocytes, contributing to OA progression [94]. OA chondrocytes exhibit hypermethylation of the COL2A1 promoter (2.3-fold increase) and hypomethylation of the MMP-13 promoter (40% decrease) compared to healthy chondrocytes. Static compression further exacerbates these epigenetic changes, leading to sustained downregulation of COL2A1 and upregulation of MMP-13 [59]. Additionally, abnormal loading downregulates miR-140 and upregulates miR-181a (a catabolic miRNA that targets SOX9), further promoting chondrocyte dedifferentiation [19].

Together, these observations demonstrate that abnormal compressive stress drives OA not only through matrix breakdown but also by triggering oxidative stress, mitochondrial dysfunction, chronic low-grade inflammation, and cellular senescence—all of which synergize to accelerate joint degeneration.

### 4.3 Translational implications for cartilage repair

Dynamic compression has emerged as a promising preprocessing strategy for tissue-engineered cartilage constructs. Preconditioning chondrocyte-scaffold constructs with dynamic compression (1 Hz, 10% strain) for 2 weeks improves their biomechanical properties (compressive modulus increased by 70%) and matrix accumulation (sGAG content increased by 50%) compared to unconditioned constructs [95]. The in vitro injury model combining collagenase treatment and mechanical loading provides a reliable tool for studying OA pathological mechanisms and screening potential therapeutics. Wen et al. [96] established an ex vivo cartilage damage model by treating cartilage explants with collagenase (0.1 mg/mL) and mechanical loading (15% strain, 1 Hz), which recapitulates key features of OA (ECM degradation, chondrocyte apoptosis, inflammatory cytokine release). This model has been used to identify novel therapeutic targets such as TRPV4 and PIEZO1.

In clinical practice, MACI is a widely used cartilage repair technique that involves implanting autologous chondrocytes seeded on a scaffold into cartilage defects [78]. However, the efficacy of MACI is limited by the dedifferentiation of chondrocytes during in vitro culture. Mechanical stimulation of chondrocytes prior to implantation restores their chondrogenic phenotype, improving the clinical outcomes of MACI. Additionally, non-invasive mechanical stimulation therapies such as low-intensity pulsed ultrasound (LIPUS) and shockwave therapy have shown promise in OA treatment: LIPUS (1.5 MHz, 30 mW/cm^2^) reduces pain and improves joint function in OA patients by promoting cartilage repair and inhibiting inflammation [97].

## 5 Optimization of scaffold materials and culture systems

**Fig. 4:**
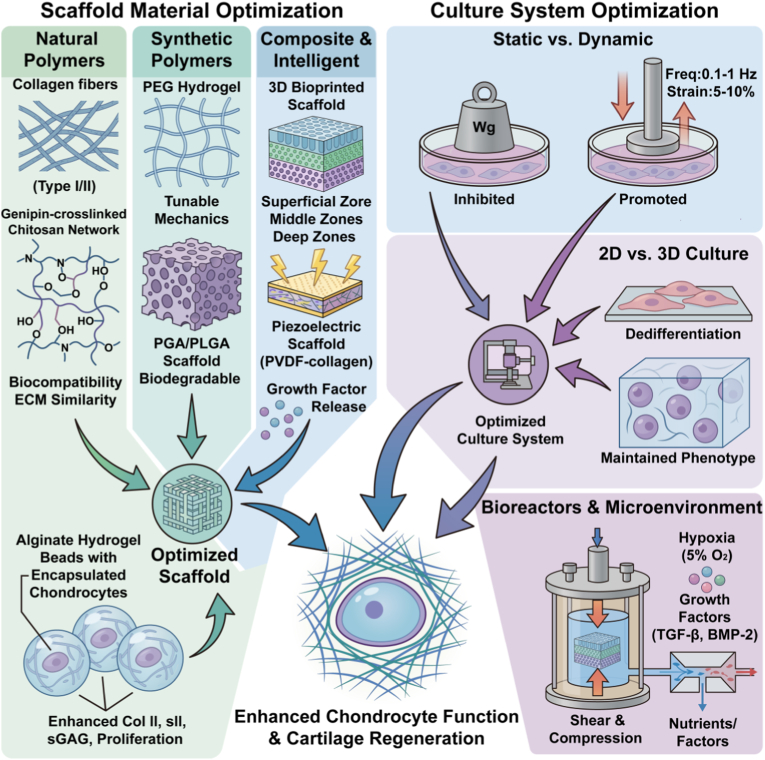
Schematic summary of scaffold material and culture system optimization strategies for enhancing chondrocyte function and promoting articular cartilage regeneration, encompassing two core domains: (1) Scaffold material optimization: three material categories are featured, including biocompatible natural polymers (collagen Type I/II, genipin-crosslinked chitosan, chondrocyte-encapsulating alginate hydrogels) that boost Col II/sGAG production and chondrocyte proliferation, synthetic polymers (PEG hydrogels with tunable mechanics, biodegradable PGA/PLGA scaffolds) with customizable properties, and composite/intelligent scaffolds (3D bioprinted zonal scaffolds mimicking native cartilage architecture, piezoelectric PVDF-collagen scaffolds, controlled growth factor release systems) integrating multi-functional characteristics for optimized performance; (2) Culture system optimization: key refinements include the superiority of dynamic loading (0.1–1 Hz frequency, 5–10% strain) over static culture for functional enhancement, 3D culture over 2D culture for maintaining chondrocyte phenotype, and integrated bioreactor systems combined with microenvironmental controls (5% O_2_ hypoxia, TGF-β/BMP-2 supplementation) for further optimization. Collectively, the synergistic integration of optimized scaffold materials and culture conditions enhances chondrocyte function and facilitates efficient articular cartilage regeneration.

The efficacy of compressive stress-mediated chondrocyte regulation is highly dependent on scaffold materials and culture systems, which must recapitulate the in vivo mechanical and biochemical microenvironment of articular cartilage. Significant progress has been made in optimizing scaffold materials (natural polymers, synthetic polymers, composites) and culture systems (static vs. dynamic, 2D vs. 3D, bioreactors) to enhance mechanical signal transduction and chondrocyte function (Fig. 4).

### 5.1 Scaffold material optimization

Scaffold materials for cartilage tissue engineering must meet three key criteria: biocompatibility (support cell adhesion, proliferation, and differentiation), mechanical properties (match the compressive modulus of articular cartilage, ∼0.1–10 MPa), and degradation rate (match ECM synthesis, ∼6–12 months) [17].

#### 5.1.1 Natural polymers

Collagen-based scaffolds, particularly type I and type II collagen, are widely used due to their excellent biocompatibility and similarity to the natural ECM. Genipin-crosslinked chitosan-collagen scaffolds improve mechanical stability by regulating crosslinking density: crosslinking with 0.5% genipin increases the compressive modulus by 2.3-fold compared to uncrosslinked scaffolds and promotes chondrocyte proliferation by 40% [98]. Collagen-chitosan composite scaffolds also enhance ECM retention, as chitosan inhibits MMP activity and reduces matrix degradation [99].

Alginate, a natural polysaccharide derived from brown algae, maintains the chondrocyte phenotype and exhibits unique matrix separation characteristics (cell-associated matrix, CM; far matrix, FRM), providing an ideal model for studying matrix metabolism under compressive stress. Alginate scaffolds with a porosity of 80% support chondrocyte adhesion and proliferation, and dynamic compression (1 Hz, 10% strain) increases sGAG content in alginate-encapsulated chondrocytes by 50% [100]. However, alginate scaffolds have weak mechanical strength and slow degradation rate, which can be improved by blending with PGA or collagen [101].

#### 5.1.2 Synthetic polymers

Polyethylene glycol (PEG) hydrogels are synthetic polymers with tunable mechanical properties and degradation rates. By precisely regulating crosslinking density, PEG hydrogels can control cell deformation and mechanotransduction: hydrogels with a crosslinking density of 10% allow 15% cell deformation under compressive stress, promoting chondrogenesis, while hydrogels with a crosslinking density of 30% restrict cell deformation, inhibiting matrix synthesis [102]. PEG hydrogels functionalized with RGD (arginine-glycine-aspartic acid) peptides enhance integrin-mediated cell adhesion and improve mechanical signal transduction [100].

Polyglycolic acid (PGA) and poly(lactic-co-glycolic acid) (PLGA) are biodegradable synthetic polymers with high porosity and mechanical strength. PGA-alginate composite scaffolds enhance ECM retention by 30% compared to pure PGA scaffolds, as alginate reduces MMP-mediated matrix degradation [101]. PLGA scaffolds loaded with TGF-β3 and seeded with MSCs exhibit improved chondrogenesis under dynamic compression, with sGAG content increased by 60% compared to unloaded scaffolds [71].

#### 5.1.3 Composite and intelligent scaffolds

Multifunctional composite scaffolds integrating natural and synthetic polymers, growth factors, and intelligent responsive elements have emerged as the next generation of cartilage tissue engineering scaffolds. Collagen-chitosan-growth factor sustained-release scaffolds balance mechanical strength, biocompatibility, and degradation rate: these scaffolds release TGF-β1 in a controlled manner (50% release over 4 weeks) and promote chondrocyte proliferation by 50% and matrix synthesis by 70% under dynamic compression [103].

Intelligent responsive scaffolds, which change their properties in response to mechanical stimuli, are designed to enhance mechanical signal transduction. For example, piezoelectric scaffolds composed of polyvinylidene fluoride (PVDF) and collagen generate electrical signals in response to compressive stress, which synergize with mechanical signals to promote chondrogenesis. PVDF-collagen scaffolds increase Col II expression by 2.5-fold and sGAG content by 60% compared to collagen scaffolds under dynamic compression [17].

3D bioprinting technology enables the fabrication of bionic scaffolds with precise microstructures that mimic the zonal architecture of articular cartilage. Mellor et al. [18] used 3D bioprinting to fabricate collagen-agarose scaffolds with superficial, middle, and deep zones, each with distinct pore sizes (100 μm, 200 μm, 300 μm) and mechanical properties. Dynamic compression of these bionic scaffolds promotes zonal-specific chondrocyte responses, with superficial zone cells showing enhanced lubrication-related gene expression and deep zone cells showing increased matrix synthesis.

### 5.2 Culture system optimization

#### 5.2.1 Static and dynamic culture

Static compression generally inhibits chondrocyte metabolism: static pressure of 3 MPa reduces collagen I/II mRNA expression by 40%–50% and proteoglycan synthesis by 30% in chondrocytes [93]. In contrast, dynamic compression shows parameter-dependent regulatory effects: appropriate frequency (0.1–1 Hz), strain (5%–10%), and duration (4–8 days) significantly promote cell proliferation, GAG, and Col II synthesis [104]. Chondrocytes encapsulated in PEG hydrogels have upregulated both anabolic (COL2A1, ACAN) and catabolic (MMP-1, MMP-13) genes under intermittent loading (1 Hz, 10% strain, 2 h/day), while continuous loading (1 Hz, 10% strain, 24 h/day) only upregulates anabolic genes and downregulates catabolic genes. This difference may be due to the recovery period provided by intermittent loading, which allows cells to adapt to mechanical stimuli [105].

#### 5.2.2 2D and 3D culture

2D culture systems fail to recapitulate the in vivo 3D microenvironment of chondrocytes, leading to dedifferentiation and reduced matrix synthesis. 3D culture systems, such as hydrogels, scaffolds, and spheroids, effectively maintain the chondrocyte phenotype by simulating ECM interactions and mechanical cues [106]. 3D embedded culture of chondrocytes in collagen-agarose hydrogels increases Col II expression by 2.3-fold and sGAG content by 50% compared to 2D culture. Additionally, 3D culture enhances the mechanosensitivity of chondrocytes: dynamic compression induces a 60% higher increase in anabolic gene expression in 3D-cultured chondrocytes compared to 2D-cultured cells [103].

#### 5.2.3 Bioreactor technology

Mechanical bioreactors simulate the in vivo mechanical environment of the joint, enhancing the physiological relevance of in vitro culture. Shahin and Doran [104] developed a mechanobioreactor that exerts simultaneous shear and compression to simulate the rolling action of articular joints. This bioreactor improves the physiological relevance of the culture system, with chondrocytes showing a 40% increase in sGAG synthesis and 30% increase in compressive modulus compared to single compressive loading. Microfluidic bioreactors, which integrate microfluidic technology with mechanical loading, enable dynamic matching of nutrient supply and load application. These bioreactors simulate the continuous fluid flow in the joint cavity, providing chondrocytes with nutrients and removing waste products while applying compressive stress [18].

#### 5.2.4 Synergistic regulation with microenvironmental factors

The effects of compressive stress on chondrocytes are synergistically regulated by microenvironmental factors such as hypoxia, inflammatory factors, and growth factors. Hypoxic environment (5% O_2_) enhances the chondrogenic response of MSCs to dynamic compression: 5% O_2_ combined with 10% strain, 1 Hz compression increases COL2A1 expression by 2.5-fold compared to normoxic environment (21% O_2_) [107]. IL-1β and 40 kPa cyclic compression synergistically promote the expression of OA-related genes (IL-8, COX-2) in chondrocytes, mimicking the inflammatory microenvironment of OA [57]. Growth factors such as TGF-β3 and BMP-2 synergize with compressive stress to enhance chondrogenesis: TGF-β3 (10 ng/mL) combined with dynamic compression increases sGAG content by 70% compared to compression alone [89].

A key implication is that abnormal compression should be viewed as a mechano-inflammatory network organizer. Mechanical overload can increase surface friction, compromise lubrication, intensify oxidative injury, and heighten inflammatory responsiveness, thereby creating a feed-forward cycle in which structural damage and adverse biomechanics perpetuate one another. Recent OA-oriented intervention studies targeting viscosupplementation/tribosupplementation, lubricating particles, and staged nano-liposomal systems are therefore conceptually relevant to compressive-stress biology because they attempt to interrupt this cycle at the level of force dissipation, boundary lubrication, and inflammatory control [[108], [109], [110], [111]].

### 5.3 Mechanotransmission of compressive mechanical cues by scaffolds and culture systems

Scaffold design, 3D culture configuration, and bioreactor systems are not merely general supportive platforms, but key mechanotransmission units that convert macroscopic compressive loading into cell-scale physical signals, including local strain, pericellular matrix (PCM) deformation, interstitial fluid pressurization, and cytoskeletal tension. These microenvironmental mechanical cues directly regulate integrin activation, calcium influx, MAPK signaling, and YAP/TAZ-mediated nuclear mechanotransduction, thereby determining whether chondrocytes exhibit anabolic or catabolic responses to compression [68,112,113] (Table 2).

**Table 2:** Scaffold and culture-system features that most directly determine how compressive cues are transmitted to chondrocytes.

Design feature	Why it matters for compression transmission	Representative implementation	Current limitation	Key cross-study comparison insights	Ref
Compressive modulus and viscoelastic recovery	Sets local strain amplitude and whether loading is buffered or concentrated	Collagen/chitosan, PEG, and composite hydrogels with tuned crosslinking	Matching cartilage stiffness without overconstraining cells remains difficult	**Horizontal comparison:** Soft collagen hydrogels (0.2–0.5 MPa) enable more efficient mechanical transfer and superior matrix production than stiff PEG scaffolds (>5 MPa). Viscoelastic gels with rapid recovery enhance anabolic signaling ∼2–3-fold relative to elastic rigid scaffolds across multiple studies.	[[114], [115], [116]]
Poroelastic permeability and fluid pressurization	Controls hydrostatic component, solute transport, and recovery after loading	Alginate-, agarose-, or porous composite-based systems	Often underreported, which hinders comparison of nominally similar regimens	**Horizontal comparison:** High-porosity scaffolds (>80%) enhance sGAG deposition by ∼40–50% via improved fluid pressurization. Studies lacking permeability/porosity data show <30% concordance in mechanical response outcomes, highlighting poor comparability.	[[117], [118], [119]]
Ligand presentation/PCM mimicry	Determines integrin engagement and focal-adhesion signaling under compression	RGD-functionalized PEG; collagen-rich matrices	Biochemical support does not guarantee physiologic force transfer	**Horizontal comparison:** Moderate RGD density (50–100 μmol/g) boosts ACAN upregulation ∼2–3-fold compared to low density. Collagen-based matrices preserve native mechanosignaling more effectively than PEG-RGD, yielding higher Col II retention across independent platforms.	[[120], [121], [122]]
Lacuna-like cell confinement	Maintains rounded morphology and modulates actin tension and YAP/TAZ activity	3D embedded culture and cell-encapsulating hydrogels	2D expansion still causes dedifferentiation before loading begins	**Horizontal comparison:** Cells in 3D lacuna-like environments show 40–60% lower YAP nuclear localization than 2D monolayers, with markedly stronger mechano-induced anabolism. Cellular roundness >0.8 predicts 3–4-fold higher mechanosensitivity across culture systems.	[[123], [124], [125]]
Zonal or osteochondral architecture	Distributes load heterogeneously across depth and improves physiologic relevance	3D bioprinted zonal scaffolds and osteochondral models	Manufacturing complexity and long-term validation are limited	**Horizontal comparison:** Gradient zonal scaffolds improve cell viability and matrix deposition by ∼30–40% relative to homogeneous scaffolds. Integration of a subchondral bone layer enhances force transmission but introduces high variability in stiffness across studies, limiting direct comparison.	[[126], [127], [128]]
Multimodal loading plus real-time sensing	Reproduces compression together with shear, hypoxia, inflammation, or readout sensors	Mechanobioreactors and cartilage-on-a-chip platforms	Low throughput and lack of standardized endpoints	**Horizontal comparison:** Combined compression + shear (1–5 dyne/cm^2^) consistently enhances chondrogenic markers by 20–50% vs. compression alone. Inflammatory readouts from organ-chip systems differ 2–3-fold in sensitivity from explant models, severely limiting cross-study quantification.	[112,129,130]

#### 5.3.1 Scaffold-mediated transmission of compressive cues

The compressive modulus, viscoelasticity, and poroelasticity of scaffolds dominate the efficiency of compressive stress transfer to chondrocytes. Soft matrices (0.2–0.5 MPa) enable sufficient cell deformation to initiate physiological mechanosensing, whereas overly stiff matrices (>5 MPa) buffer strain and blunt mechanotransduction [100,114]. Viscoelastic scaffolds with rapid stress relaxation better sustain PCM deformation and FAK activation under dynamic compression [114]. High-porosity structures amplify hydrostatic pressure and enhance TRPV4/PIEZO1 channel activation [117,118]. RGD or collagen II functionalization strengthens integrin–matrix coupling and improves the transmission of compressive deformation to intracellular signaling [102,120,121]. Zonal bioprinted scaffolds further distribute compressive stress heterogeneously along the tissue depth, recapitulating native mechanical gradients and driving region-specific mechanotransduction [123,126,127].

#### 5.3.2 3D culture governs cellular sensing of compressive stress

2D culture induces chondrocyte spreading, elevated actin tension, and YAP/TAZ nuclear localization, which abolish physiological compression sensing [40,124]. In contrast, 3D lacuna-like confinement maintains a rounded cell morphology, reduces cytoskeletal tension, and restores mechanosensitivity to compressive loading [124,125,127]. A cellular roundness value > 0.8 predicts 3–4-fold stronger compression-induced calcium signaling and anabolic gene expression [124]. By mimicking native PCM mechanics, 3D culture enables faithful translation of compressive cues into intracellular signals [120,122].

#### 5.3.3 Bioreactor systems deliver physiologically relevant compressive mechanical cues

Single-axis compression cannot fully mimic in vivo joint mechanics. Bioreactors that apply simultaneous shear and compression reproduce rolling–sliding motion more realistically and enhance integrin engagement, calcium signaling, and matrix synthesis compared with compression alone [104,129]. Microfluidic bioreactors couple dynamic compression with controlled interstitial flow, optimizing mechanical signal presentation and nutrient transport [18,130]. Real-time monitoring modules allow standardized delivery of compressive cues and quantitative evaluation of mechanotransduction [130].

### 5.4 Current limitations and future directions

Despite significant advances, scaffold materials and culture systems still face several challenges: (1) Scaffold properties are difficult to balance: pure collagen scaffolds have weak mechanical strength, high crosslinking density PEG hydrogels have insufficient elasticity, and alginate scaffolds have mismatched degradation rates [100]; (2) Compression parameters lack standardization: dynamic compression frequency (0.001–1 Hz), strain (1%–50%), and pressure (1–10 MPa) vary greatly across studies, leading to difficulties in horizontal comparison [12]; (3) Mechanotransduction mechanisms under different scaffold-load combinations are unclear: the differences and interactions of signaling pathways (e.g., integrin-calcium-MAPK) in different scaffolds remain incompletely defined [100]; (4) Physiological load simulation is limited: most studies focus on single compressive load, while in vivo joints experience shear-compression synergistic loading [104]; (5) Clinical translation is insufficient: most studies remain in in vitro experiments, lacking long-term in vivo verification [17].

Future optimization should focus on: (1) Developing multifunctional composite scaffolds using collagen-chitosan-growth factor sustained-release systems, PEG crosslinking modification, and 3D bioprinting technology to balance mechanical strength, biocompatibility, and degradation rate [103]; (2) Establishing personalized compression parameter databases by integrating load response data of different scaffolds and cell sources (normal/OA patients) using big data and machine learning [18]; (3) Deepening the research on signal mechanisms under different load-scaffold combinations, focusing on core targets such as integrin, calcium, and MAPK pathways [100]; (4) Improving mechanical bioreactors to simulate shear-compression synergistic loading and dynamic nutrient-load matching using microfluidic technology [104]; (5) Conducting long-term in vivo experiments on large animal models to verify the repair effect of optimized systems on cartilage defects [97]; (6) Exploring the synergistic effect of growth factor sustained release, hypoxia, and mechanical loading to construct a comprehensive regulatory system closer to in vivo conditions [107].

## 6 Auxiliary mechanisms of compressive stress stimulation

**Fig. 5:**
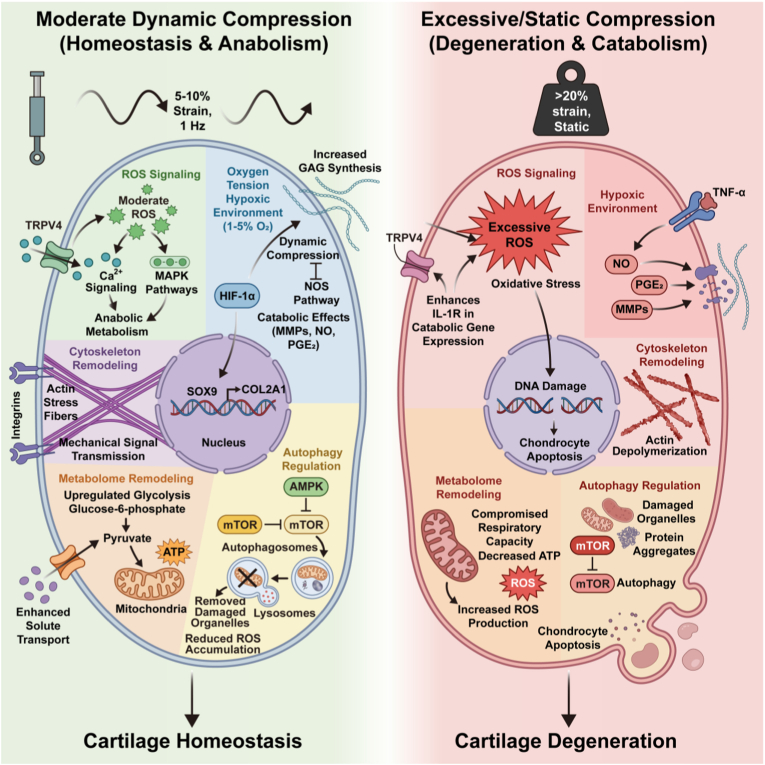
Schematic contrast of chondrocyte intracellular mechanisms and functional outcomes in response to moderate dynamic compression versus excessive/static compression, with a color-coded legend (green = beneficial/homeostatic; red = detrimental/catabolic) denoting functional effects: Left panel (moderate dynamic compression, 5–10% strain, 1 Hz) triggers homeostatic and anabolic responses, including activation of TRPV4/Ca^2+^ and MAPK signaling pathways, cytoskeleton remodeling via integrins/actin fibers, metabolome reprogramming (upregulated glycolysis and ATP production), HIF-1α-mediated suppression of pathological NOS pathways and promotion of SOX9/COL2A1 expression (for matrix synthesis), and AMPK-driven autophagy (mTOR inhibition) to mitigate ROS accumulation by clearing damaged organelles, collectively sustaining cartilage homeostasis; Right panel (excessive/static compression, >20% static strain) induces degenerative and catabolic cascades, characterized by excessive ROS generation (causing oxidative stress, DNA damage and chondrocyte apoptosis), TRPV4-mediated upregulation of IL-1R expression coupled with TNF-α signaling to drive catabolic factors (NO, PGE_2_, MMPs), metabolome disruption (impaired respiratory capacity and reduced ATP levels), dysregulated autophagy (mTOR dysfunction and damaged protein/organelle accumulation), cytoskeleton depolymerization, and hypoxic amplification of pathological signaling, ultimately leading to cartilage degeneration. (For interpretation of the references to color in this figure legend, the reader is referred to the Web version of this article.)

In addition to the core mechanotransduction pathways discussed above, compressive stress regulates chondrocyte function through auxiliary mechanisms involving ROS signaling, oxygen tension, cell cytoskeleton remodeling, and metabolome remodeling. These auxiliary mechanisms integrate with core pathways to form a comprehensive regulatory network (Fig. 5).

### 6.1 ROS signaling

Compressive stress induces ROS production in chondrocytes, which plays a dual role in mechanical responses. Mild dynamic compression (5% strain, 1 Hz) increases ROS production by 20%–30% in chondrocytes, which acts as a second messenger to activate calcium signaling and MAPK pathways, promoting anabolic metabolism [131]. Cyclic compression increases IL-1R expression by inducing ROS in 3D-cultured ATDC5 cells, enhancing cell sensitivity to IL-1β. This process is regulated by TRPV4, as TRPV4 agonists inhibit ROS production and catabolic gene expression, while antagonists enhance them [26]. In contrast, excessive compressive stress (20% strain, static loading) induces excessive ROS production (2–3-fold increase), leading to oxidative stress, DNA damage, and chondrocyte apoptosis. The balance between ROS production and scavenging determines the functional outcome of mechanical stress: moderate ROS levels promote cartilage homeostasis, while excessive ROS levels drive degeneration [14].

### 6.2 Oxygen tension

Oxygen tension is a critical microenvironmental factor that modulates the effects of compressive stress on chondrocytes [107]. Articular cartilage is a hypoxic tissue, with oxygen tension ranging from 1% to 5% in the deep zone to 10%–15% in the superficial zone. The activities of NO, PGE_2_, and MMP induced by TNF-α in hypoxic environment (5% O_2_) are significantly higher than those in normoxic environment (21% O_2_). Dynamic compression reduces this catabolic effect by inhibiting the NOS pathway, with hypoxic environment enhancing the protective effect of compression. Oxygen tension also affects the amplitude of GAG synthesis: dynamic compression increases sGAG content by 60% in hypoxic environment compared to 30% in normoxic environment. This regulation is mediated by hypoxia-inducible factor 1α (HIF-1α), which is upregulated by both hypoxia and dynamic compression and promotes chondrogenesis by activating SOX9 and COL2A1.

### 6.3 Cytoskeleton remodeling

Mechanical loads induce reversible remodeling of the chondrocyte actin cytoskeleton, which plays a key role in mechanotransduction. Mechanical compression and hydrostatic pressure induce actin cytoskeleton reorganization in chondrocytes in agarose: dynamic compression leads to the formation of stress fibers, while static compression causes actin depolymerization. The actin cytoskeleton acts as a mechanical linker between integrins and intracellular signaling molecules, transmitting mechanical signals from the cell surface to the nucleus. Inhibition of actin polymerization with cytochalasin D abrogates compression-mediated calcium influx and MAPK activation, confirming the critical role of cytoskeleton remodeling in mechanotransduction [132]. Additionally, the microtubule cytoskeleton is involved in compressive stress responses: Primary human chondrocytes respond to compression with phosphoproteomic signatures that include microtubule activation, as inhibition of microtubule polymerization reduces compression-mediated matrix synthesis [21].

### 6.4 Metabolome remodeling

Compressive stress alters the metabolomic profile of chondrocytes, regulating energy metabolism and nutrient transport [133]. Shear and compression have distinct effects on the metabolomic profile of SW1353 chondrocytes: compression upregulates metabolites involved in glycolysis (glucose-6-phosphate, pyruvate) and downregulates metabolites involved in oxidative phosphorylation (ATP, NADH), while shear stress shows the opposite effect. Dynamic compression enhances the interstitial transport of glucose-like solutes in articular cartilage by 40%, improving nutrient supply to chondrocytes [134]. Additionally, joint load changes chondrocyte respiratory function: injurious loading compromises mitochondrial respiratory capacity, reducing ATP production by 30% and increasing ROS production [135]. The metabolomic remodeling induced by compressive stress provides energy and substrates for matrix synthesis, highlighting the integration of mechanical signaling with cellular metabolism.

### 6.5 Autophagy regulation

Autophagy, a conserved cellular process that degrades and recycles damaged organelles and proteins, is regulated by compressive stress and contributes to chondrocyte survival [2]. Moderate dynamic compression (10% strain, 1 Hz) upregulates autophagy in chondrocytes, as evidenced by increased LC3-II expression and autophagosome formation. Autophagy promotes chondrocyte survival by removing damaged mitochondria and reducing ROS accumulation. In contrast, excessive compressive stress inhibits autophagy, leading to accumulation of damaged organelles and enhanced apoptosis [136,137]. The regulation of autophagy by compressive stress is mediated by the AMPK-mTOR pathway: dynamic compression activates AMPK, which inhibits mTOR and induces autophagy. Inhibition of autophagy abrogates the protective effects of dynamic compression, confirming its role in mechanical stress responses.

## 7 Conclusion and outlook

This review provides a comprehensive overview of the molecular mechanisms, cell-specific responses, and translational applications of compressive stress-driven chondrocyte mechanotransduction. We have explicitly distinguished mechanisms supported by direct, causal evidence from those that are associative or preliminary to provide a clear critical perspective. Key insights include: (1) Compressive stress initiates a cascade of mechanosensing, signal transduction, and transcriptional regulation via integrins, calcium channels, MAPK pathways, and epigenetic modifications; (2) Articular cartilage regional heterogeneity and cell type-specific responses highlight the need for personalized mechanical intervention strategies; (3) Moderate dynamic compression promotes cartilage repair by enhancing anabolic metabolism and inhibiting inflammation, while abnormal loading drives OA progression; (4) Optimization of scaffold materials and culture systems enhances mechanical signal transduction and improves the efficacy of tissue-engineered cartilage; (5) Auxiliary mechanisms such as ROS signaling, oxygen tension, and autophagy integrate with core pathways to regulate chondrocyte function.

Despite significant progress, several critical challenges remain: (1) In vitro models lack physiological relevance, failing to simulate multi-axial loading, dynamic inflammatory gradients, and cartilage-bone interface interactions; (2) Loading parameters are unstandardized, leading to contradictory results and hindering cross-study comparison; (3) Pathway crosstalk and epigenetic regulation mechanisms are incompletely understood; (4) Clinical translation is limited by the lack of non-invasive delivery systems and personalized treatment protocols.

Future research should prioritize the following directions: (1) Decipher multi-pathway synergistic networks using multi-omics technologies (transcriptomics, proteomics, metabolomics) to identify key regulatory nodes; (2) Establish personalized mechanical parameter databases integrating patient-specific factors (age, gender, disease severity) via multi-center studies; (3) Develop bionic models using microfluidic technology, 3D bioprinting, and dynamic microenvironmental factors to improve in vivo relevance; (4) Optimize mechanical-biological combined therapies, including intelligent load-responsive scaffolds, non-invasive mechanical stimulation, and molecular targeting; (5) Conduct large animal experiments and clinical prospective studies to verify the safety and efficacy of standardized parameters; (6) Explore the effects of age, gender, and comorbidities (e.g., obesity, diabetes) on chondrocyte mechanical responses to develop precision medicine strategies.

In conclusion, compressive stress is a double-edged sword in cartilage biology: physiological loading maintains tissue homeostasis, while abnormal loading drives degeneration. Deciphering the complex mechanisms of compressive stress-mediated chondrocyte regulation and optimizing mechanical intervention strategies will provide novel molecular targets and clinical translation schemes for cartilage repair and OA intervention, ultimately improving the quality of life for patients with cartilage-related diseases.

## CRediT authorship contribution statement

**Yongbing Mou:** Investigation, Writing – original draft. **Tingting Tian:** Methodology, Project administration. **Peng Wang:** Writing – original draft. **Xia Wang:** Writing – original draft. **Wei Li:** Data curation. **Wenfei Tang:** Validation. **Yehong Wang:** Data curation. **Dong Zhu:** Conceptualization, Funding acquisition. **Yong Huang:** Conceptualization, Writing – review & editing. **Xiao Huang:** Conceptualization, Funding acquisition, Project administration, Writing – review & editing.

## Declaration of competing interest

The authors declare that they have no known competing financial interests or personal relationships that could have appeared to influence the work reported in this paper.

## Data Availability

No data was used for the research described in the article.
